# Analysis of Current Status on a New Public Health Nutrition Service Pattern in China: A Nutrition Outpatient Clinic-Based Study

**DOI:** 10.1155/2018/6143738

**Published:** 2018-08-09

**Authors:** Yunpeng Jin, Xiaolin Li

**Affiliations:** ^1^Division of Cardiology, The Fourth Affiliated Hospital of Zhejiang University School of Medicine, N1 Shangcheng Road, Yiwu 322000, Zhejiang, China; ^2^Division of Nutrition, The Fourth Affiliated Hospital of Zhejiang University School of Medicine, N1 Shangcheng Road, Yiwu 322000, Zhejiang, China

## Abstract

**Background:**

Nutrition outpatient clinics were launched in some hospitals as a new pattern of public health nutrition service in recent years in China. The aim of this study was to review and analysis demographics and consultation spectrum in a single nutrition outpatient clinical center in China.

**Methods:**

The retrospective study was performed in the nutrition outpatient clinical center launched by a comprehensive teaching hospital in Yiwu, Zhejiang Province (China). 1014 patients attending the clinic from August 2015 to February 2018 were included. The clinical records including relevant history and baseline data were reviewed and analyzed.

**Results:**

Majority of the patients (41.9%) came to our clinical center for nutrition consultation of healthy dietary services, 32.1% for malnutrition, 6.7% for diabetes, 6.3% for neoplasms, 5.3% for digestive system diseases, and the last 7.6% for hypertension, hematologic diseases, thyroid diseases, and so on. More minor patients came for healthy dietary services compared with the population (P<0.001), and, on the contrary, more adult patients came for malnutrition service, especially obesity (P<0.05), while more elderly patients came for consultation services of diabetes, neoplasms, digestive system diseases, and hypertension (P<0.05).

**Conclusion:**

Our study provided important data of the new public health nutrition service pattern in China which has never been reported yet. It indicated the huge demand of nutrition outpatient clinic, especially nutrition consultation services of healthy diet and malnutrition; further studies about the validity of the new pattern in improving public health nutrition status are expected in the future.

## 1. Introduction

More and more nutrition health issues occurred in China recent years as economy develops. The Fourth National Nutrition and Health Survey showed that China is facing the dual challenges of nutritional deficiencies and overnutrition [1, 2]. The dramatic increase in nutrition-related chronic diseases such as obesity, hyperlipidemia, diabetes, cardio-cerebral vascular disease, and cancer posed a heavy burden on society [3, 4]. The demand of public health nutrition services has increased in China. Therefore, nutrition outpatient clinics were launched in some hospitals in recent years.

As a new pattern of public health nutrition service, the nutrition outpatient clinics in China were supported by nutrition physicians, not only physicians but also nutritionists [5, 6]. According to several investigation researches in China, about 12% of Chinese hospitals have launched nutrition outpatient clinics. However, the data about the nutrition outpatient clinics has never been reported yet.

In our nutrition outpatient clinical center, thousands of patients have been attended in the past more than two years. The aim of this study was to review and analyze demographics and consultation spectrum in a single nutrition outpatient clinical center in China.

## 2. Methods

### 2.1. Study Subjects

All consecutive new patients seen in a comprehensive teaching hospital (The Fourth Affiliated Hospital of Zhejiang University School of Medicine) nutrition clinical center from August 2015 to February 2018 were included in this retrospective study. The clinical records were reviewed and the demographics of the patients, their height, weight, Body Mass Index (BMI), and the cause of attending were analyzed. According to the types of nutrition-related issues, nutrition services included healthy dietary services, malnutrition, diabetes, neoplasms, digestive system diseases, hypertension, and so on.

### 2.2. Ethical Aspects

Ethical approval for publication of this data was obtained from the Ethics Committee of The Fourth Affiliated Hospital of Zhejiang University School of Medicine.

### 2.3. Anthropometric Measurements

All patients used the same devices, which were calibrated at the time of measurement. The height and weight of adolescents were measured with thin clothes (shorts and T-shirts) and without shoes. Height was measured to the nearest 0.1 cm with a free-standing stadiometer mounted on a rigid tripod (GMCS-I, Xindong Huateng Sports Equipment Co. Ltd., Beijing, China). Fasting body weight was measured to the nearest 0.1 kg on a digital scale (RGT-140, Weighing Apparatus Co. Ltd, Changzhou Wujin, China). Body mass index (BMI) was calculated by body weight (kg) / [body height (m)]^2^. Thinness, overweight, and obesity were defined according to the special cutoffs of BMI<18.5 kg/m^2^, BMI≥24 kg/m^2^, and BMI≥28 kg/m^2^, respectively, for the Chinese adult population [7]. Thinness, overweight, and obesity in children and adolescents population were defined according to age- and sex-specific BMI cutoffs developed for Chinese children and adolescents [8]. Malnutrition was defined by BMI z-score in infancy, related to the 2006 World Health Organization growth references.

### 2.4. Statistical Analysis

All categorical variables are expressed as proportions. Chi-square tests were used for intergroup comparisons of categorical variables. A p value <0.05 was considered significant, and all p values are 2-sided. Statistical analyses were performed with SPSS software version 19 (IBM/SPSS, Armonk, NY USA 2012).

## 3. Results

From August 2015 to February 2018, 1014 patients had been seen in the nutrition clinical center as new cases. The age ranged from 4 months to 92 years old. Proportion of female patients was a little bit more than male. A large proportion of adult patients (18-64 years old) came to our clinic for nutrition services, and minor patients (0- <18 years old) accounted for less than one-third, while the number of elderly patients (≥65 years old) was quite small. About a half of the patients were normal weight; underweight and obesity patients accounted for about one-fifth which was about a double of the overweight patients. Most of them were Han Chinese came from urban area and received education for 7-12 years. The demographics of the patients are shown in Table 1.

Majority of the patients (41.9%) came to our clinical center for nutrition consultation of healthy dietary services, 32.1% for malnutrition, 6.7% for diabetes, 6.3% for neoplasms, 5.3% for digestive system diseases, and the last 7.6% for hypertension, hematologic diseases, thyroid diseases, and so on.

Healthy dietary services consisted of nutrition consultation of common diet (34.7%), growth and development (3.4%), maternal nutrition (1.8%), preparation of pregnant (0.7%), and others (1.3%) such as preparation of college entrance examination and breastfeeding.

Malnutrition patients were divided into two groups: undernutrition (11.4%) and overnutrition (20.7%). The undernutrition group included thinness (9.5%), child nutrition disorders (1.5%), and deficiency diseases (0.5%). The overnutrition group consisted of overweight (6.9%) and obesity (13.8%).

More minor patients came for healthy dietary services compared with the population (P<0.001), and, on the contrary, more adult patients came for malnutrition service, especially obesity (P<0.05), while more elderly patients came for consultation services of diabetes, neoplasms, digestive system diseases, and hypertension (P<0.05). The consultation spectrum of the patients is shown in Table 2.

No significant sex differences were found in the patients who came for nutrition services. About a half of the patients who came for healthy dietary services were minors, while most of the patients who came for malnutrition services were adults (P<0.001). More adult patients came due to overnutrition compared with undernutrition (P<0.01). Differences of demographics between main nutrition services are shown in Table 3.

## 4. Discussion

Along with the increasing incidence of nutrition-related chronic diseases such as obesity, hyperlipidemia, diabetes, cardio-cerebral vascular disease, cancer, and public awareness, there is a growing demand of public health nutrition service for nutrition-related issues in China in recent years.

A concerted effort was made in the mid-1980s to foster clinical nutrition in major hospitals throughout the country by programs directed at medical graduates and nursing and kitchen staff in China [9]. Traditionally, clinical nutritionists without prescription are only responsible for in-patient nutritional assessment and consultation as teamwork members with physicians of related specialties. Only a few comprehensive teaching hospitals established independent nutrition clinics as the new pattern of nutrition service, due to few physicians willing to accept nutrition professional training to work as nutrition physicians; from then on, the new public health nutrition service patterns were launched.

Our study showed that thousands of patients had been seen in the nutrition clinic as new cases in the past more than two years which indicated tremendous demand.

The population served by our nutrition outpatient clinic is extensive: age of patients ranged from 4 months to 92 years old. A large proportion of adult patients came to our clinic for nutrition services; on the contrary, minor patients only accounted for less than one-third and the number of elderly patients is quite small. However, population aging is becoming increasingly prominent in developing countries. The need for nutritional assessment and intervention is particularly crucial in elderly people due to the high incidence of chronic illness and malnutrition [10]. Similarly, the infant and young child population suffered more and more nutrition issues such as feeding practices, less breastfeeding, anemia, and protein energy malnutrition [11]. So, we believe that the awareness of nutrition issues for infant and young child and elderly population needs to be raised urgently.

Majority of the patients came to our clinic for healthy dietary services. The healthy dietary services is dietary nutrition consultation for all stages of healthy population without diseases. The main services were nutrition consultation of common diet, growth and development, maternal nutrition, and preparation of pregnancy.

The common diet nutrition consultation accounted for the largest proportion which may due to the unreasonable food consumption patterns. For adult, some studies indicated that Chinese food consumption patterns and eating and cooking behaviors changed dramatically with urbanization, for instance, the increasing consumption of energy from fats, the high level of sodium consumption, and the high sodium-potassium ratios [12]. For child population, many parents who came to our clinic complain that their children were picky eaters, in addition to high intakes of total fat and saturated fat, low intakes of fruits and vegetables were the most common problems. Some research indicated that the low intakes of fruits and vegetables are associated with inadequate intakes of vitamin A, vitamin C, and dietary fiber [13]. For infant and young child population, the early initiation of breastfeeding knowledge and practices of caregivers was poor. For example, most common problems are the insufficient time of exclusive breastfeeding and failing to add iron-rich or iron-fortified foods timely. Unreasonable food consumption patterns were considered as the reasons for many nutrition-related chronic diseases; therefore, the Chinese Nutrition Society revised the Food Guide Pagoda for Chinese Residents 2016 [14], after that the dietary counseling services provided followed.

Besides healthy dietary services, the second highest ranked nutrition service is about malnutrition. The prevalence of overweight and obesity is increasing worldwide and even developing countries are beginning to experience this trend in the last two decades [15]. It is common to see problems of underweight, stunting, and micronutrient deficiencies even with the prevalence of overweight and obesity increasing [16]. Our study showed that the number of overnutrition patients is significantly greater than undernutrition. As the rapid economic growth, overweight and obesity have become a major public health problem in China. Because of earlier experiences with long-term poverty and famine, the Chinese traditionally believe that fatness is a sign of happiness and abundance. These traditional beliefs have not kept pace with the socioeconomic changes that have led to a more secure food supply, overconsumption, and energy imbalance [17]. Therefore, we believe that the government should pay more attention to the overnutrition problems and take effective policy measures to curb the trend towards overweight and obesity in China. In addition, the proportion of overweight patients is significantly lower than obesity which may resulted from insufficient awareness of the early recognition of obesity and the harm to obesity.

Some other patients came to our clinic for various disease-related nutritional counseling. The main diseases were diabetes, neoplasms, digestive system diseases, and so on. Research has shown that diet is the cornerstone of treatment in diabetes and dietary advice should be tailored to the individual and their circumstances [18]. Nutritional management of the cancer patient cannot be ignored, given the well-established links between malnutrition and mortality, postoperative complications, toxicity of radiation, chemotherapy, and quality of life [19]. It is indicated that the comprehensive clinical medical background knowledge of nutrition physician is often required given the diversity of the clinical nutrition-related diseases in our clinic.

The limitation of our research is that it is a retrospective study in a single center. A nonrandom and unrepresentative sample limits the generalizability of our study findings. However, almost all patients in the local area came to our center. So we believe that our conclusions are credible and meaningful.

## 5. Conclusion

The present study provided important data of the new public health nutrition service pattern in China which has never been reported yet. It indicated the huge demand of nutrition outpatient clinic, especially nutrition consultation services of healthy diet and malnutrition; further studies about the validity of the new pattern in improving public health nutrition status are expected in the future.

## Figures and Tables

**Table 1:** Demographics of the patients attending the nutrition clinical center (n=1014).

Demographics	No. (%) of patients
Sex	
Male	483(47.6)
Female	531(52.4)
Age (years)	
0- <18	281(27.7 )
18-64	669(66.0)
≥65	64(6.3)
BMI categories	
Underweight	222(21.9)
Normal weight	455(44.9)
Overweight	114(11.2)
Obesity	223(22.0)
Region	
Urban	947(93.4)
Rural	67(6.6)
Education (years)	
0	119(11.7)
0-6	121(11.9)
7-12	609(60.1)
>12	165(16.3)
Ethnic group (nationalities)	
Han	989(97.5)
Others	25(2.5)

BMI, body mass index.

**Table 2:** Nutrition services in patients attending the nutrition clinical center.

Nutrition services	Patients	Female	P Value^(a)^	Age 0- <18	P value^(a)^	Age 18-64	P value^(a)^	Age ≥65	P value^(a)^
Healthy Dietary Services	425(41.9)	229(53.9)	0.599	199(46.8)	<0.001 ^(b)^	218(51.3)	<0.001 ^(b)^	8(1.9)	<0.001 ^(b)^
Common diet consultation	352(34.7)	181(51.4)	0.759	159(45.2)	<0.001 ^(b)^	185(52.6)	<0.001 ^(b)^	8(2.3)	0.004 ^(b)^
Growth and development consultation	35(3.5)	13(37.1)	0.076	35(100.0)	<0.001 ^(b)^	0(0.0)	<0.001 ^(b)^	0(0.0)	0.266 ^(b)^
maternal consultation	18(1.8)	18(100.0)	<0.001 ^(b)^	0(0.0)	0.006 ^(b)^	18(100.0)	0.001 ^(b)^	0(0.0)	0.621
Preparation of pregnant consultation	7(0.7)	7(100.0)	<0.001 ^(b)^	0(0.0)	0.200	7(100.0)	0.103	0(0.0)	1.000
others	13(1.3)	10(76.9)	0.096	5(38.5)	0.153	8(61.5)	0.772	0(0.0)	1.000
Malnutrition	326(32.1)	157(48.2)	0.186	70(21.5)	0.118	247(75.8)	0.001 ^(b)^	9(2.8)	0.014 ^(b)^
Undernutrition	116(11.4)	65(56.0)	0.454	33(28.4)	0.867	77(66.4)	0.931	6(5.2)	0.630
Thinness	96(9.5)	58(60.4)	0.131	15(15.6)	0.010 ^(b)^	75(78.1)	0.016 ^(b)^	6(6.3)	0.981
Child nutrition disorders	15(1.5)	5(33.3)	0.193	15(100.0)	<0.001 ^(b)^	0(0.0)	<0.001 ^(b)^	0(0.0)	0.617
Deficiency diseases	5(0.5)	2(40.0)	0.674	3(60.0)	0.136	2(40.0)	0.345	0(0.0)	1.000
Overnutrition	210(20.7)	92(43.8)	0.024 ^(b)^	37(17.6)	0.002 ^(b)^	170(81.0)	<0.001 ^(b)^	3(1.4)	0.002 ^(b)^
Overweight	70(6.9)	31(44.3)	0.191	15(21.4)	0.254	54(77.1)	0.055	1(1.4)	0.118
Obesity	140(13.8)	61(43.6)	0.051	22(15.7)	0.002 ^(b)^	116(82.9)	<0.001 ^(b)^	2(1.4)	0.018 ^(b)^
Diabetes	68(6.7)	33(48.5)	0.621	0(0.0)	<0.001 ^(b)^	53(77.9)	0.043 ^(b)^	15(22.1)	<0.001 ^(b)^
Neoplasms	64(6.3)	42(65.6)	0.039 ^(b)^	0(0.0)	<0.001 ^(b)^	52(81.2)	0.012 ^(b)^	12(18.8)	<0.001 ^(b)^
Digestive system diseases	56(5.3)	25(44.6)	0.260	4(7.1)	<0.001 ^(b)^	43(76.8)	0.095	9(16.1)	0.005 ^(b)^
Hypertension	16(1.6)	11(68.8)	0.217	0(0.0)	0.009 ^(b)^	12(75.0)	0.598	4(25.0)	0.017 ^(b)^
Hematologic diseases	12(1.2)	5(41.7)	0.566	4(33.3)	0.747	6(50.0)	0.358	2(16.7)	0.178
Thyroid diseases	10(1.0)	9(90.0)	0.023 ^(b)^	2(20.0)	0.735	8(80.0)	0.509	0(0.0)	1.000
Atherosclerosis	8(0.8)	4(50.0)	1.000	0(0.0)	0.116	4(50.0)	0.456	4(50.0)	0.001 ^(b)^
Respiration disorders	8(0.8)	4(50.0)	1.000	2(25.0)	1.000	5(62.5)	1.000	1(12.5)	0.410
Hyperuricemia	6(0.6)	0(0.0)	0.032 ^(b)^	0(0.0)	0.196	6(100.0)	0.102	0(0.0)	1.000
Hyperlipidemia	5(0.5)	4(80.0)	0.377	0(0.0)	0.331	5(100.0)	0.173	0(0.0)	1.000
Kidney diseases	4(0.4)	3(75.0)	0.626	0(0.0)	0.580	4(100.0)	0.306	0(0.0)	1.000
Mental disorders	3(0.3)	2(66.7)	1.000	0(0.0)	0.565	3(100.0)	0.555	0(0.0)	1.000
Endocrine system diseases	3(0.3)	3(100.0)	0.251	0(0.0)	0.565	3(100.0)	0.555	0(0.0)	1.000

Values are presented as number (%). (a) Values for proportion of patients in each group were compared with the population (n=1014) using chi-squared test and Fisher exact test. (b) P-value <0.05.

**Table 3:** Differences of demographics between main nutrition services.

Variable	Healthy Dietary Services (n=425)	Malnutrition (n=326)	P Value^(a)^	Undernutrition (n=116)	Overnutrition (n=210)	P Value^(a)^
Sex						
Female	229 (53.9)	157(48.2)	0.120	65(56.0)	92(43.8)	0.086
Age (years)						
0- <18	199 (46.8)	70(21.5)	<0.001 ^(b)^	33(28.4)	37(17.6)	0.023 ^(b)^
18-64	218(51.3)	247(75.8)	<0.001 ^(b)^	77(66.4)	170(81.0)	0.003 ^(b)^
≥65	8(1.9)	9(2.8)	0.422	6(5.2)	3(1.4)	0.073

Values are presented as number (%). (a) Values for proportion of patients in each group were compared with each other using chi-squared test and Fisher exact test. (b) P-value <0.05.

## Data Availability

The data used to support the findings of this study are available from the corresponding author upon request.
